# Associated organs and system with COVID-19 death with information of organ support: a multicenter observational study

**DOI:** 10.1186/s12879-023-08817-5

**Published:** 2023-11-20

**Authors:** Ryuichi Nakayama, Naofumi Bunya, Takashi Tagami, Mineji Hayakawa, Kazuma Yamakawa, Akira Endo, Takayuki Ogura, Atsushi Hirayama, Hideo Yasunaga, Shuji Uemura, Eichi Narimatsu

**Affiliations:** 1 Department of Emergency Medicine, Sapporo Medical University School of Medicine, 291, Minami 1-jo Nishi 16-chome, Chuo-ku, Sapporo, Hokkaido, 060-8543 Japan; 2 Department of Emergency and Critical Care Medicine, Nippon Medical School Musashikosugi Hospital, Tokyo, Japan; 3 Department of Clinical Epidemiology and Health Economics, School of Public Health, The University of Tokyo, Tokyo, Japan; 4 Department of Emergency Medicine, Hokkaido University Hospital, Hokkaido, Japan; 5 Department of Emergency Medicine, Osaka Medical and Pharmaceutical University, Osaka, Japan; 6 Trauma and Acute Critical Care Center, Tokyo Medical and Dental University Hospital, Tokyo, Japan; 7 Department of Emergency Medicine and Critical Care Medicine, Tochigi Prefectural Emergency and Critical Care Centre, Imperial Foundation Saiseikai Utsunomiya Hospital, Tochigi, Japan; 8 Department of Social Medicine, Graduate School of Medicine, Public Health, Osaka University, Osaka, Japan

**Keywords:** COVID-19, Death, Organ dysfunction

## Abstract

**Background:**

The organ dysfunction that is associated with death in COVID-19 patients has not been determined in multicenter epidemiologic studies. In this study, we evaluated the major association with death, concomitant organ dysfunction, and proportion of multiple organ failure in deaths in patients with COVID-19, along with information on organ support.

**Methods:**

We performed an observational cohort study using the Japanese multicenter research of COVID-19 by assembling a real-world data (J-RECOVER) study database. This database consists of data on patients discharged between January 1 and September 31, 2020, with positive SARS-CoV-2 test results, regardless of intensive care unit admission status. These data were collected from the Diagnosis Procedure Combination and electronic medical records of 66 hospitals in Japan. The clinician identified and recorded the organ responsible for the death of COVID-19.

**Results:**

During the research period, 4,700 patients with COVID-19 were discharged from 66 hospitals participating in the J-RECOVER study; of which, 272 patients (5.8%) from 47 institutions who died were included in this study. Respiratory system dysfunction (87.1%) was the leading association with death, followed by cardiovascular (4.8%), central nervous (2.9%), gastrointestinal (2.6%), and renal (1.1%) dysfunction. Most patients (96.7%) who died of COVID-19 had respiratory system damage, and about half (48.9%) had multi-organ damage. Of the patients whose main association with death was respiratory dysfunction, 120 (50.6%) received mechanical ventilation.

**Conclusion:**

This study showed that although respiratory dysfunction was the most common association with death in many cases, multi-organ dysfunction was associated with death due to COVID-19.

**Supplementary Information:**

The online version contains supplementary material available at 10.1186/s12879-023-08817-5.

## Introduction

Coronavirus disease 2019 (COVID-19), caused by severe acute respiratory syndrome coronavirus 2 (SARS-CoV-2), is an infectious disease with pandemic status, with more than 278 million confirmed cases and nearly 5.4 million deaths worldwide from the outbreak at the end of 2019 to the end of December 2021 [1]. COVID-19 is most characterized by respiratory symptoms and pneumonia [2], but it is also known to induce a variety of dysfunctions and clinical manifestations in organs other than the respiratory tract, which is recognized as a multisystem inflammatory syndrome, but the extent to which it contributes to death is unknown [3, 4].

The organ dysfunction that associated with death in COVID-19 patients has not been determined in multicenter epidemiologic studies. Only three single-center studies and one multicenter case series have reported the organs and system associated with death due to COVID-19. Zhang et al. reported 82 patients with COVID-19 who died between January 11, 2020, and February 10, 2020, at the Renmin Hospital of Wuhan University (RHWU). In this report, 75.6% of patients who died had comorbidities. Respiratory dysfunction was the most common (69.5%), but only 4.8% of patients received invasive ventilation. The second most common cause of death was sepsis, but the organ affected by the infection was unknown. In most cases (90.2%), two or more organs were damaged [5]. Wang et al. similarly reported 77 patients with COVID-19 who died from February 1, 2020, to March 7, 2020, in the Eastern Branch of RHWU. Respiratory dysfunction was the most common (87.0%), and 72.7% of the patients who died had comorbidities, but only 9.1% of the patients received invasive ventilation [6]. These studies were limited as only a few patients received invasive mechanical ventilation due to limited medical resources caused by the pandemic. Ketcham et al. similarly reported that respiratory dysfunction was the most common [7], while Elezkurtaj et al.’s multicenter case series (26 cases) reported that multiorgan failure and septic shock were more common [8]. However, there has been no large-scale cohort study.

This study aimed to determine the organs and system associated with death and proportion of multiple organ failure in deaths in patients dying from COVID-19, along with information on organ support, such as mechanical ventilation.

## Methods

### Study design

We performed an observational cohort study using the Japanese multicenter research of COVID-19 by assembling real-world data (J-RECOVER) database. The details of the study design and protocol of the J-RECOVER were previously described elsewhere [9]. Data were collected from 66 hospitals in Japan. This study was approved by the Ethics Committee of Sapporo Medical University (Approval number: 322 − 245) on February 12, 2021. This study was conducted in compliance with the principles of the Declaration of Helsinki, approved by the ethics committees of each participating hospital, and registered at the UMIN Clinical Trials Registry (UMIN000047056). As this was a retrospective observational study using existing medical information, informed consent from each patient was waived by the Ethics Committee of Sapporo Medical University, and the patients and their relatives were guaranteed the opportunity to opt out of participation after information about the current study was published and disclosed on the hospital’s website (https://nms-kosugi-eccm.com/covid19-joint-research/).

### Study participants

The J-RECOVER study database contains data from patients across all age groups who were discharged between January 1 and September 30, 2020, who tested positive for SARS-CoV-2, regardless of intensive care unit (ICU) admission status. The database excludes cases treated on an outpatient basis without hospital admission as well as out-of-hospital cardiac arrest cases that were confirmed dead upon arrival at the emergency department.

The study focused on COVID-19 patients who died during the study period. Patients for whom information on survival and death was not available or for whom organ dysfunction that associated with death was not recorded were excluded.

### Data collection

In the present study, existing clinical information of patients was obtained from the diagnosis procedure combination (DPC) data. The diagnostic group classification system based on DPC was introduced in Japan in 2002, and currently, more than 1,600 acute care hospitals participate in the system and submit DPC data to the Ministry of Health, Labour and Welfare [10]. Clinical information was acquired anonymously from the DPC data using a specialized software called DPC Hash application. DPC data included sex, date of birth, height, weight, current pregnancy, dates of admission and discharge, outcome at discharge, main disease name, comorbidities at the time of admission, all medical and surgical treatments, and records of all prescribed drugs and devices. Each diagnosis was defined based on the International Classification of Diseases, 10th revision (ICD-10) codes. To assess comorbidity, the Charlson comorbidity index was calculated from the ICD-10 codes for each comorbidity diagnosis [11]. To ensure the validity of the coding, the physician in charge was to log the name of the diagnosis in the medical record.

In addition, laboratory data and information necessary to solve the research problem, which were not available in the DPC data, were obtained from the medical records. Additional clinical information included the date of symptom onset, Glasgow Coma Scale score, blood pressure, pulse rate, respiratory rate, body temperature, and race. Survival time was defined as the period from symptom onset until death. When the onset of symptoms was unknown, it was substituted with the date of positive SARS-CoV-2 test.

### Collecting information on organ dysfunction primarily associated with death

The clinician identified and recorded the organ responsible for death in COVID-19 patients. To identify organ dysfunction that was the primary association with death, the following codes were used to record organ dysfunction in deceased patients: (1) Respiratory system, (2) Cardiovascular system, (3) Central nervous system, (4) Thromboembolic event, (5) Hemorrhagic event, (6) Renal system, (7) Gastrointestinal system, (8) Liver, (9) Biliary tract, (10) Pancreas, and 11) Other. One code for organ dysfunction was selected as the primary association with death, and all codes for organ dysfunction associated with death were recorded. The questionnaire utilized for data collection can be found in Supplementary Table 1 (English version).

### Data analysis

A descriptive analysis was performed to identify the epidemiological and clinical characteristics of the patients who died from COVID-19. Continuous variables were shown as median and interquartile range (IQR) and were compared using the Mann–Whitney U test. Categorical variables were shown as percentages in different categories and were compared using the Chi-square test. Statistical significance was set at *P* < 0.05. Data analysis was performed using SPSS Statistics version 25 (IBM Corp., Armonk, NY, USA) and R version 4.1.2 (R Foundation for Statistical Computing, Vienna, Austria).

## Results

**Fig. 1:**
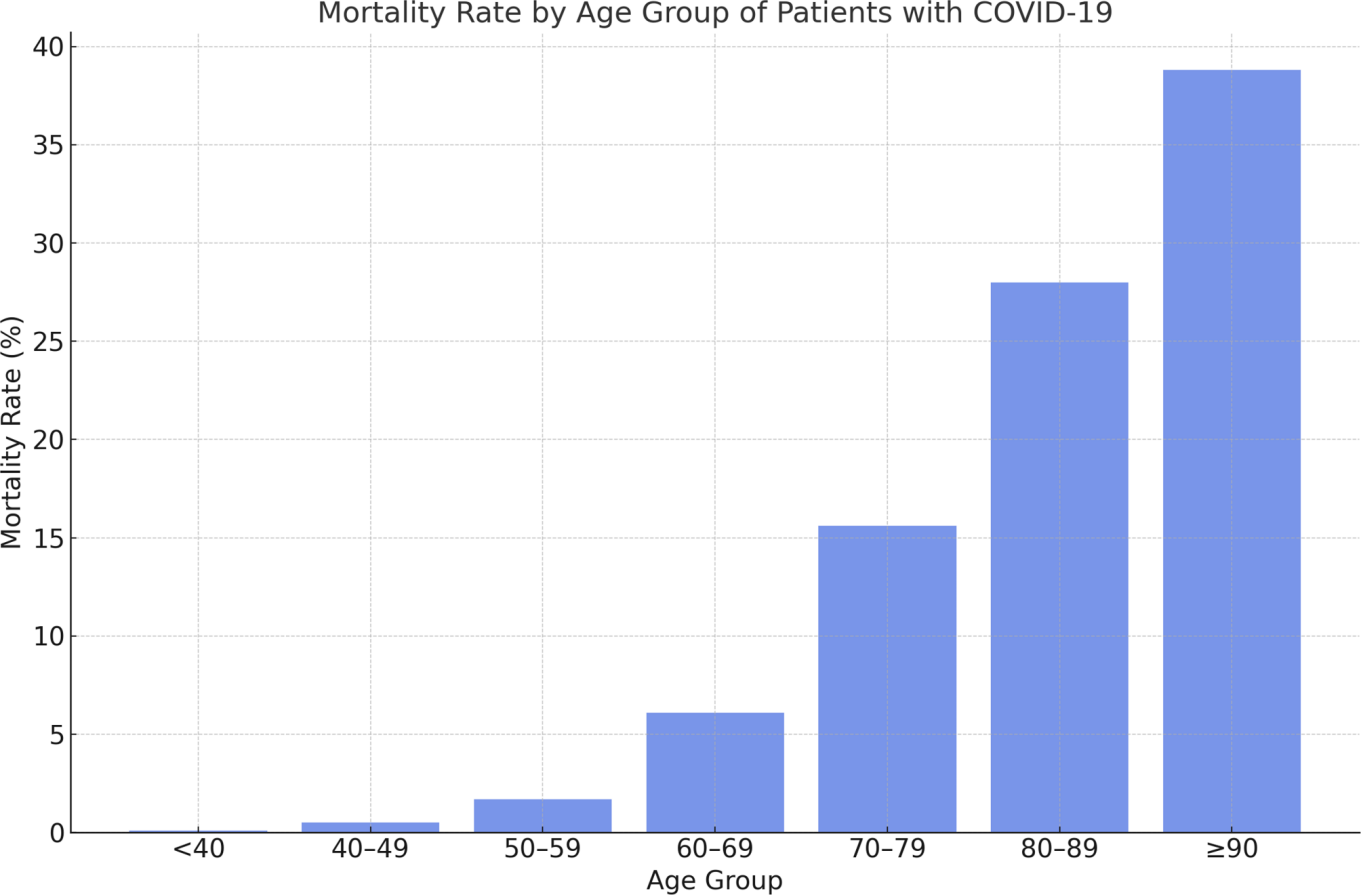
Mortality by age group of patients with COVID-19

From January 1 to September 31, 2020, 4,700 patients with COVID-19 were discharged from 66 institutions participating in the J-RECOVER study, of which, 272 patients (5.8%) from 47 institutions who died were included, and there were no exclusions in this study. The overall mortality rate of the study participants was 5.8%, and the mortality rate increased with age (Fig. 1). The characteristics of the patients with COVID-19 who died are shown in Table 1. The median age of the study subjects age was 78 years (IQR 72, 84), 253 (93.0%) were 60 years or older, most were men (72.8%), 160 patients (61.5%) were treated in the ICU, and the median survival time was 22 days. Laboratory data on admission showed median values of 10.5 mg/dl for C-reactive protein (CRP), 394 U/L for lactate dehydrogenase (LDH), 819.9 ng/mL for ferritin, and 2.2 µg/mL for D-dimer, which were high, and 0.28 ng/mL for procalcitonin, which was normal.

**Table 1:** Characteristics of dead patients with COVID-19 at admission

Patient characteristics	n = 272
Age, years	78 [72–84]
Sex, Male, n (%)	198/272 (72.8)
Body Mass Index, kg/m^2^	22.8 [20.5–26.0]
Charlson index	0 [0–1]
Pregnancy, n (%)	0/272 (0)
Race Japanese, n (%)	269/270 (99.6)
Intensive care unit admission, n (%)	160/260 (61.5)
Pneumonia identified by radiological examination, n (%)	259/270 (95.9)
Days from onset to admission	6 [3–12]
Survival time (from disease onset to death), days	22 [16–33]
Glasgow Coma Scale	15 [13–15]
Mean blood pressure, mmHg	93.3 [82–105]
Pulse rate, /min	90 [78–105]
Respiratory rate, /min	22 [18–26]
SpO_2_, %	95 [91–97]
Body temperature, ˚C	37.3 [36.7–38.0]
White blood cell, ×10^3^ /µl	6.4 [4.5–9.8]
Neutrophil, %	88.0 [80.3–92.3]
Lymphocyte, %	5.0 [10.6–16.7]
Monocyte, %	4.1 [2.3–6.7]
Eosinophil, %	0.0 [0.0–0.3]
Basophil, %	0.2 [0.0–0.3]
Hematocrit, %	37.6 [32.6–41.4]
Platelet, ×10^4^ /µl	16.6 [12.6–22.0]
Albumin, g/dl	2.9 [2.4–3.3]
Total bilirubin, mg/dl	0.6 [0.4–0.8]
AST, U/L	47.0 [32.0–68.0]
ALT, U/L	27.0 [18.0–44.5]
BUN, mg/dl	24.2 [17.0–35.0]
Creatinine, mg/dl	0.96 [0.72–1.49]
LDH, U/L	394 [293–537]
Na, mEq/L	138 [134–141]
K, mEq/L	4.1 [3.7–4.5]
CRP, mg/dl	10.5 [5.2–17.3]
Procalcitonin, ng/mL	0.28 [0.13–1.21]
KL-6, U/mL	390 [265–782]
Ferritin, ng/mL	819.9 [386.0–1383.8]
PT-INR	1.06 [1.00–1.13]
APTT, sec	34.7 [31.0–39.2]
D-dimer, µg/ml	2.2 [1.2–4.3]
Fibrinogen, mg/dl	502.5 [425.8–599.3]
HbA1c, %	6.4 [5.7–7.1]

Continuous variables were expressed as median [interquartile range]. Categorical variables were n/N (%), where N is the total number of patients with COVID-19 in the relevant data minus missing values

ALT, alanine aminotransferase; AST, aspartate aminotransferase; APTT, activated partial thromboplastin time;BUN, blood urea nitrogen; CRP, C-reactive protein; HbA1c, hemoglobin A1c; KL-6, Krebs von den Lungen-6; LDH, lactate dehydrogenase; PT-INR, prothrombin time-international normalized ratio

The analysis of organ dysfunction in patients with COVID-19 is presented in Table 2. Respiratory system dysfunction (87.1%) represented the leading association with death, followed by cardiovascular (4.8%), central nervous (2.9%), gastrointestinal (2.6%), and renal (1.1%) system dysfunction. The higher mortality rate due to respiratory system dysfunction with increasing age is presented in Supplementary Table 2. Most patients (96.7%) who died of COVID-19 had respiratory system damage, and about half (48.9%) exhibited damage in two or more organs and/or systems. Of the patients whose primary association with death other than respiratory dysfunction, 26 (74.3%) had respiratory dysfunction and received mechanical ventilation. Most patients (88.6%) whose primary association with death was other than respiratory dysfunction had more than one organ failure.

**Table 2:** Dysfunction of organs and system of dead patients with COVID-19.

	N = 272
**Organs and system most associated with death**	
Respiratory system	237 (87.1)
Cardiovascular system	13 (4.8)
Central nervous system	8 (2.9)
Gastrointestinal system	7 (2.6)
Renal system	3 (1.1)
Hemorrhagic event	1 (0.4)
Biliary tract	1 (0.4)
Other	2 (0.7)
Thromboembolic event	0 (0.0)
Liver	0 (0.0)
Pancreas	0 (0.0)
**Dysfunction of organs and system**	
Respiratory system	263 (96.7)
Renal system	85 (31.3)
Cardiovascular system	69 (25.4)
Central nervous system	27 (9.9)
Liver	25 (9.2)
Thromboembolic event	23 (8.5)
Hemorrhagic event	22 (8.1)
Gastrointestinal system	14 (5.1)
Biliary tract	6 (2.2)
Other	5 (1.8)
Pancreas	2 (0.7)
**Number of Dysfunction of organs and system**	
1	140 (51.5)
2	62 (22.8)
3	34 (12.5)
4	17 (6.3)
≥5	19 (7.0)

Data were expressed as n (%)

Of the patients whose primary association with death was respiratory dysfunction, 120 (50.6%) received mechanical ventilation, with a median age of 76 years; and 117 (49.4%) had no mechanical ventilation, with a median age of 81 years, showing a significant difference in age (*P* < 0.001) (Tables 3 and 4). Supplementary Table 3 showed that the mechanical ventilation rate in deaths primarily associated with respiratory dysfunction decreased as the age category increased.

**Table 3:** Organ dysfunction and supportive care in cases where the respiratory system was the primary association with death and in other cases

Organs and system as primary association with death		High flow nasal cannula	Mechanical ventilation	V-V ECMO	V-A ECMO
**Respiratory system**	237	4	120	15	0
Dysfunction of only respiratory system	135	3	57	3	0
Dysfunction of two or more organs, including respiratory system	102	1	63	12	0
**Organ dysfunction other than respiratory system**	35	1	26	13	1
Dysfunction of only one system	4	0	1	1	0
Dysfunction of two or more organs, excluding respiratory system	5	1	4	2	0
Dysfunction of two or more organs, including respiratory system	26	0	21	10	1

V-A ECMO, veno-arterial Extracorporeal Membrane Oxygenation; V-V ECMO, veno-venous extracorporeal membrane oxygenation

**Table 4:** Characteristics of deaths with and without ventilation when respiratory system is the primary association with death

Respiratory dysfunction (n = 237)	Mechanical ventilation (n = 120)	No mechanical ventilation (n = 117)	*P* value
Age, years	76 [69–81]	81 [75–88]	< 0.001
Sex, Male	91 (75.8)	79 (67.5)	0.20
Body Mass Index	24.1 [22.0–26.8]	21.5 [19.3–24.3]	< 0.001
Intensive care unit admission	110 (91.7)	22 (18.8)	< 0.001
Survival time (from disease onset to death), days	27 [19–37]	17 [12–24]	< 0.001

Continuous variables were expressed as median [interquartile range]. Categorical variables were n/N (%), where N is the total number of patients with COVID-19 in the relevant data minus missing values. Continuous variables were compared using the Mann–Whitney U test. Categorical variables were shown as percentages in different categories and were compared using the Chi-square test

Supplementary Table 4 presents specific diagnoses for each organ derived from the DPC data, identifying the primary association with death excluding respiratory dysfunction. Congestive heart failure and gastrointestinal bleeding were both the most frequently observed, with three cases each. Cases where the diagnosis could not be determined from the DPC data were categorized as “unknown”. Moreover, Supplementary Table 5 offers a comparison of inflammatory responses based on respiratory system vs. non-respiratory system as primary association with death. Specifically, levels of Procalcitonin and Ferritin were significantly higher in the non-respiratory system group than in the respiratory system group.

## Discussion

This was the first multicenter nationwide study to identify organ dysfunction as primary association with death in patients with COVID-19. This study highlights that although respiratory system dysfunction was the most common association with death, multiple organ dysfunction was also associated with death in many cases. In addition, this study suggests that the mechanical ventilatory rate decreased with increasing age in deaths due to respiratory failure.

Similar to the previous studies [5–7, 12, 13], we found that respiratory dysfunction was the most common association with death in COVID-19 patients. We believe that the results of this large cohort study will contribute to the generalization compared to a single-center study. The reason for not receiving mechanical ventilation in COVID-19 deaths primarily associated with respiratory dysfunction may be related to age. Although the higher rate of invasive mechanical ventilation compared to the previous studies [5, 6] suggests that more intensive care resources were allocated in Japan, the shortage of medical resources due to the pandemic also occurred [14], and it is possible that medical resources such as mechanical ventilation were allocated to younger patients. This hypothesis is supported by the fact that a survey conducted in Japan revealed that non-medical and medical personnel largely agreed on the triage process: allocation prioritizing those most likely to recover on ventilators, allocation prioritizing treatment of the young, and allocation prioritizing those who can live longer after ventilatory treatment [15]. A similar phenomenon of lower rates of ventilatory use with increasing age was observed in nationwide data from Germany, where the rate of ventilatory use was less than 1% in people over 90 years of age [16]. The small number of oxygen therapy sessions with a high-flow nasal cannula (HFNC) may be attributed to the fact that HFNC was not recommended during this study period because it might increase the risk of viral transmission [17].

Similar to the study by Zhang et al. [5], we found that the second leading association with death in COVID-19 was cardiovascular system dysfunction, and that there was also a high incidence of multiple organ damage, which is consistent with the case series by Elezkurtaj et al. [8]. A review by Chang et al. reported an increased risk of death in patients with COVID-19 with concomitant cardiac dysfunction [18]. The pathogenesis of SARS-CoV-2 induced cardiac damage remains not fully understood, but several mechanisms have been postulated. These include the direct role of the ACE2 receptor, through which SARS-CoV-2 binds and penetrates various cell types, leading to potential myocardial inflammation; a hyperimmune response characterized by cytokine storms and systemic inflammation; and the activation of the TLR4 pathway, which enhances ACE2 expression and subsequently facilitates viral entry, resulting in hyperinflammation and potential organ injuries. Myocardial damage may also stem from the profound alteration of endothelial homeostasis, leading to manifestations such as oedema, microvascular inflammation, and atherothrombosis [19]. Recent evidence on cardiac magnetic resonance imaging suggests that myocardial angiopathy and its complications might be primary mechanisms of acute myocardial injury, with myocarditis being a rarer occurrence [20]. These may induce new cardiac pathologies (myocardial infarction, heart failure, etc.) or exacerbate underlying cardiovascular diseases, and are thought to contribute to increased mortality.

In patients whose organ disfunction primarily associated with death was not respiratory system, congestive heart failure and gastrointestinal bleeding were the most common specific diagnoses according to the DPC data (Supplementary Table 4). One possible reason for multiple organ damage is that in patients with acute respiratory distress syndrome, inflammation induced by lung injury can cause biotrauma and damage to other organs as well [21]. In addition, COVID-19 was reported to cause cytokine storms characterized by elevated serum levels of pro-inflammatory cytokines and multi-organ damage, which is known as Multisystem Inflammatory Syndrome in Adults (MIS-A) [3, 22]. A comparison between the respiratory and non-respiratory groups primarily associated with death revealed a trend toward a stronger inflammatory response in the non-respiratory group (Supplementary Table 5). This trend may suggest the involvement of MIS-A in COVID-19 cases. In this study cohort, thromboembolic events were reported in 8.5% of patients, but no deaths were reported with a thromboembolic event as the primary association. This could be due in part to racial differences from other countries, but the mechanism is unclear and requires further study [23].

Although previous studies have shown that older age is associated with higher mortality [15, 24, 25], no studies have shown which organ damage contributes to death by age group. In all age groups, deaths from respiratory disorders were the most common cause of death, especially in patients over 70 years of age, where respiratory disorders were the primary association with death in 90.3% of patients.

The strength of this study is that the large, multicenter cohort strengthens the generalization that the most common cause of death in COVID-19 is respiratory failure, and that death is often due to multiple organ damage.

This study had several limitations. Firstly, the messenger RNA vaccine against SARS-CoV-2 was introduced in Japan in February 2021 [26], and none of the study participants had been vaccinated at that time. Further studies are needed on the vaccinated population. Secondly, the J-RECOVER database used for this study did not include postmortem examination data but collected clinical information on organ dysfunction. Thirdly, clear data criteria for organ dysfunction were not established within this database. Decisions regarding data collection items for the registry were finalized in late 2020. At that time, criteria for COVID-19 multi-organ failure [7] or MIS-A was not well-defined. While the CDC has since presented criteria for MIS-A [27], the standards for organ failure remain ambiguous [28]. Consequently, drawing from single-center studies [5, 6], we devised a code table. Attending physicians, primarily specializing in emergency or intensive care and well-versed in evaluating impaired organs, then identified the organ predominantly associated with death, along with other affected organs. This constraint affects the interpretation of this study’s results. Fourthly, this study primarily aimed to elucidate injured organ in deceased patients with COVID-19. However, it is essential to acknowledge that the interpretation of causal relationships between COVID-19 and organ dysfunction leading to death is constrained. We did not account for the temporal progression of laboratory findings, the course of comorbidities, the responsiveness to therapies, or potential iatrogenic effects of treatments. Consequently, drawing definitive conclusions regarding causality remains challenging. Fifthly, the specific distribution of medical resources in hospitals during the peak of the COVID-19 pandemic might have influenced our findings. This unique allocation could introduce bias in the data, potentially affecting the overall interpretation of the study results. Finally, the present study did not follow the patients once they were discharged from the hospital and did not catch deaths from post-acute sequelae of COVID-19 or the long-term prognosis after discharge for COVID-19 infection. Further research is needed to investigate the long-term prognosis of COVID-19 post-discharge, to identify organ dysfunction as a cause of death after vaccine introduction, and to confirm these topics in other ethnic groups besides Asians.

## Conclusion

Through a multi-center nationwide observational study, this study showed that although respiratory dysfunction was the most common association with death, in many cases, multi-organ dysfunction was associated with death due to COVID-19.

### Electronic supplementary material

Below is the link to the electronic supplementary material.

Supplementary Material 1

## Data Availability

The datasets used and/or analyzed during the current study are available from the corresponding author upon reasonable request and with permission of the J-RECOVER group. Once the paper is published, all data generated or analyzed during this study will be included in the published article and its Additional files. Please contact the corresponding author to request the survey data set.

## References

[1] Health organization World Health Organization. Coronavirus disease (COVID-19) Weekly epidemiological update on COVID-19-2021; https://www.who.int/emergencies/diseases/novel-coronavirus-2019/situation-reports (accessed 1 Jan. 2022).

[2] Stokes EK, Zambrano LD, Anderson KN (2020). Coronavirus Disease 2019 case surveillance - United States, January 22–May 30, 2020. MMWR Morb Mortal Wkly Rep.

[3] Patel P, DeCuir J, Abrams J, Campbell AP, Godfred-Cato S, Belay ED (2021). Clinical characteristics of multisystem inflammatory syndrome in adults: a systematic review. JAMA Netw Open.

[4] Davogustto GE, Clark DE, Hardison E (2021). Characteristics associated with multisystem inflammatory syndrome among adults with SARS-CoV-2 Infection. JAMA Netw Open.

[5] Zhang B, Zhou X, Qiu Y (2020). Clinical characteristics of 82 cases of death from COVID-19. PLoS ONE.

[6] Wang K, Qiu Z, Liu J (2020). Analysis of the clinical characteristics of 77 COVID-19 deaths. Sci Rep.

[7] Ketcham SW, Bolig TC, Molling DJ, Sjoding MW, Flanders SA, Prescott HC (2021). Causes and circumstances of death among patients hospitalized with COVID-19: a retrospective cohort study. Ann Am Thorac Soc.

[8] Elezkurtaj S, Greuel S, Ihlow J (2021). Causes of death and comorbidities in hospitalized patients with COVID-19. Sci Rep.

[9] Tagami T, Yamakawa K, Endo A (2022). Japanese multicenter research of COVID-19 by assembling real-world data: a study protocol. Annals Clin Epidemiol.

[10] Hayashida K, Murakami G, Matsuda S, Fushimi K (2021). History and profile of diagnosis procedure combination (DPC): development of a real data collection system for acute inpatient care in Japan. J Epidemiol.

[11] Quan H, Li B, Couris CM (2011). Updating and validating the Charlson comorbidity index and score for risk adjustment in hospital discharge abstracts using data from 6 countries. Am J Epidemiol.

[12] Reddy DR, Cuenca JA, Botdorf J (2023). Clinical characteristics and cause of death among hospitalized decedents with Cancer and COVID-19. Mayo Clin Proc.

[13] Cannizzo JP, Chai AL, Do CT, Wilson ML, Liebler JM, Huerta LE (2023). Causes of death among medical ICU patients with Pneumonia due to COVID-19 in a safety-net hospital. Crit Care Explor.

[14] Dar M, Swamy L, Gavin D, Theodore A (2021). Mechanical-ventilation supply and options for the COVID-19 pandemic. Leveraging all available resources for a limited resource in a crisis. Ann Am Thorac Soc.

[15] Norisue Y, Deshpande GA, Kamada M (2021). Allocation of mechanical ventilators during a pandemic: a mixed-methods study of perceptions among Japanese Health Care Workers and the General Public. Chest.

[16] Ludwig M, Jacob J, Basedow F, Andersohn F, Walker J (2021). Clinical outcomes and characteristics of patients hospitalized for Influenza or COVID-19 in Germany. Int J Infect Dis.

[17] Japan ECMOnet for COVID-19. Respiratory support in covid-19-related critically ill patients. [accessed on 24th October 2023]. https://square.umin.ac.jp/jrcm/pdf/info20200312.pdf.

[18] Chang WT, Toh HS, Liao CT, Yu WL (2021). Cardiac involvement of COVID-19: a comprehensive review. Am J Med Sci.

[19] Molina-Ramos AI, Gómez-Moyano E, Rodríguez-Capitán J (2022). Myocarditis related to COVID-19 and SARS-CoV-2 vaccination. J Clin Med.

[20] Artico J, Shiwani H, Moon JC (2023). Myocardial involvement after hospitalization for COVID-19 complicated by troponin elevation: a prospective, Multicenter, Observational Study. Circulation.

[21] Meyer NJ, Gattinoni L, Calfee CS (2021). Acute respiratory distress syndrome. Lancet.

[22] Anka AU, Tahir MI, Abubakar SD (2021). Coronavirus Disease 2019 (COVID-19): an overview of the immunopathology, serological diagnosis and management. Scand J Immunol.

[23] Iba T, Connors JM, Spyropoulos AC, Wada H, Levy JH (2021). Ethnic differences in thromboprophylaxis for COVID-19 patients: should they be considered?. Int J Hematol.

[24] Levin AT, Hanage WP, Owusu-Boaitey N, Cochran KB, Walsh SP, Meyerowitz-Katz G (2020). Assessing the age specificity of Infection fatality rates for COVID-19: systematic review, meta-analysis, and public policy implications. Eur J Epidemiol.

[25] Abore K, Weldeab AB, Berasa, Amsalu Midaso T. Epidemiological and Clinical Profile of deaths due to COVID-19 among hospitalized patients in Sidama Region, Ethiopia " Global Journal of Epidemiology and Infectious Disease (2022): 69–77.

[26] Hara M, Furue T, Fukuoka M (2022). Real-world effectiveness of the mRNA COVID-19 vaccines in Japan: a case-control study. Vaccines (Basel).

[27] Centers for Disease Control and Prevention (CDC). Multisystem inflammatory syndrome in adults (MIS-A) case definition information for healthcare providers. [accessed on 24th October 2023]. https://www.cdc.gov/mis/mis-a/hcp.html.

[28] Kunal S, Ish P, Sakthivel P, Malhotra N, Gupta K (2022). The emerging threat of multisystem inflammatory syndrome in adults (MIS-A) in COVID-19: a systematic review. Heart Lung.

