# Supplementary material for: Associated organs and system with COVID-19 death with information of organ support: a multicenter observational study
Source: BMC Infect Dis. 2023 Nov 20;23:814. doi: 10.1186/s12879-023-08817-5 (PMC10662555; doi:10.1186/s12879-023-08817-5)
Supplement: Supplementary file 1 — Supplementary Material 1 [file 12879_2023_8817_MOESM1_ESM.doc]

**Supplementary material**

Supplementary Table 1.

The questionnaire used to collect data for organ dysfunction associated with death.

|  |  | Code | Free space |
| --- | --- | --- | --- |
| 1 | Choose a code number (from 1 to 11) that, in your opinion, represents the primary association with death. Even if some patients might have succumbed to multiple organ failure, please select the one you believe was the primary factor leading to death. If you select code number 11, please provide details in the space provided. |  |  |
| 2 | Select all code number (from 1 to 11) corresponding to the organs associated with the death, including what you believe to be the primary cause of death, from the options in the bottom right corner. |  |  |

Supplementary Table 2.

Dysfunction of organs and system of dead patients with COVID-19 by age group.

|  | **Age group** | | | | | | |
| --- | --- | --- | --- | --- | --- | --- | --- |
|  | **<40** | **40-49** | **50-59** | **60-69** | **70-79** | **80-89** | **≥90** |
| **Organs and system as primary association with death** | | | | | | | |
| Respiratory system | - | - | 9 | 32 | 85 | 85 | 26 |
| Cardiovascular system | - | 1 | - | 2 | 5 | 5 | - |
| Central nervous system | - | 2 | 2 | 3 | 1 | - | - |
| Gastrointestinal system | 1 | - | - | 1 | 1 | 4 | - |
| Renal system | - | - | - | - | 1 | 2 | - |
| Hemorrhagic event | - | - | 1 | - | - | - | - |
| Biliary tract | - | - | - | 1 | - | - | - |
| Other | - | - | - | - | 1 | 1 | - |
| Thromboembolic event | - | - | - | - | - | - | - |
| Liver | - | - | - | - | - | - | - |
| Pancreas | - | - | - | - | - | - | - |
| **Dysfunction of organs and system** | | | | | | | |
| Respiratory system | 1 | 2 | 12 | 36 | 93 | 93 | 26 |
| Renal system | 1 | 2 | 3 | 14 | 27 | 33 | 5 |
| Cardiovascular system | 1 | 2 | 3 | 11 | 23 | 26 | 3 |
| Central nervous system | 1 | 2 | 3 | 6 | 6 | 8 | 1 |
| Liver | 1 | 1 | - | 3 | 10 | 10 | - |
| Thromboembolic event | - | 2 | - | 7 | 5 | 9 | - |
| Hemorrhagic event | 1 | - | 3 | 7 | 6 | 4 | 1 |
| Gastrointestinal system | 1 | - | - | 2 | 4 | 7 | - |
| Biliary tract | - | 1 | - | 1 | - | 4 | - |
| Other | - | - | - | - | 1 | 2 | 2 |
| Pancreas | - | - | - | - | - | 2 | - |
| **Number of organs and system dysfunction** | | | | | | | |
| 1 | - | 1 | 7 | 16 | 50 | 50 | 16 |
| 2 | - | - | 1 | 11 | 24 | 18 | 8 |
| 3 | - | - | 2 | 5 | 8 | 17 | 2 |
| 4 | - | 1 | 1 | 2 | 9 | 4 | - |
| ≥5 | 1 | 1 | 1 | 5 | 3 | 8 | - |

Supplemental Table 3.

Proportion of invasive mechanical ventilation in COVID-19 deaths primarily associated with respiratory dysfunction.

|  | Death cases | Mechanical ventilation | Proportion of mechanical ventilation in COVID-19 deaths (%) |
| --- | --- | --- | --- |
| Total | 237 | 120 | 50.6 |
| Age group |  |  |  |
| <40 | 0 | 0 | 0.0 |
| 40–49 | 0 | 0 | 0.0 |
| 50–59 | 9 | 5 | 55.6 |
| 60–69 | 32 | 27 | 84.4 |
| 70–79 | 85 | 46 | 54.1 |
| 80–89 | 85 | 39 | 45.9 |
| ≥90 | 26 | 3 | 11.5 |

Supplemental Table 4.

Specific diagnosis for each organ as primary association with death except for respiratory dysfunction based on diagnosis procedure combination data.

| Organs and system  as primary association with death | Specific diagnosis based on  diagnosis procedure combination data | n/N (%) |
| --- | --- | --- |
| Cardiovascular system, N = 13 | Congestive heart failure | 3/13 (23.1) |
|  | Infective endocarditis | 2/13 (15.4) |
|  | Extremity peripheral artery disease | 1/13 (7.7) |
|  | Unknown | 7/13 (53.8) |
| Central nervous system, N = 8 | Stroke | 2/8 (25.0) |
|  | Acute subdural hematoma | 1/8 (12.5) |
|  | Unknown | 5/8 (62.5) |
| Gastrointestinal system, N = 7 | Gastrointestinal bleeding | 3/7 (42.9) |
|  | Peritonitis | 1/7 (14.3) |
|  | Intra-abdominal abscess | 1/7 (14.3) |
|  | Unknown | 2/7 (28.6) |
| Renal system, N = 3 | Microscopic polyangiitis | 1/3 (33.3) |
|  | Unknown | 2/3 (66.7) |
| Hemorrhagic event, N = 1 | Hemothorax | 1/1 (100.0) |
| Other, N = 2 | hyperosmolar hyperglycemic state | 1/2 (50.0) |
|  | Unknown | 1/2 (50.0) |

Supplemental Table 5.

Comparison of inflammatory responses between respiratory system and non-respiratory system groups in primary association with death

|  | Organs and system as primary association with death | |  |
| --- | --- | --- | --- |
|  | Respiratory system | Non-respiratory system | *P* value |
| CRP, mg/dl | 10.0 [5.1–17.0] | 13.4 [6.6–22.1] | 0.10 |
| Procalcitonin, ng/mL | 0.23 [0.11–0.98] | 0.51 [0.26–3.08] | 0.0091 |
| Ferritin, ng/mL | 748.5 [358.8–1236.5] | 1404.5 [689.0–2588.8] | 0.0039 |

Continuous variables were expressed as median [interquartile range] and compared using the Mann–Whitney U test.

CRP, C-reactive protein
